# Carnosine Counteracts the Molecular Alterations Aβ Oligomers-Induced in Human Retinal Pigment Epithelial Cells

**DOI:** 10.3390/molecules28083324

**Published:** 2023-04-09

**Authors:** Giuseppe Caruso, Claudia G. Fresta, Annamaria Fidilio, Francesca Lazzara, Nicolò Musso, Vincenzo Cardaci, Filippo Drago, Filippo Caraci, Claudio Bucolo

**Affiliations:** 1 Department of Drug and Health Sciences, University of Catania, 95125 Catania, Italy; 2 Unit of Neuropharmacology and Translational Neurosciences, Oasi Research Institute-IRCCS, 94018 Troina, Italy; 3 Department of Biomedical and Biotechnological Sciences, University of Catania, 95123 Catania, Italy; 4 Bio-Nanotech Research and Innovation Tower (BRIT), University of Catania, 95123 Catania, Italy; 5 Vita-Salute San Raffaele University, 20132 Milano, Italy; 6 Scuola Superiore di Catania, University of Catania, 95123 Catania, Italy; 7 Center for Research in Ocular Pharmacology-CERFO, University of Catania, 95123 Catania, Italy

**Keywords:** age-related macular degeneration, amyloid-beta oligomers, inflammation, oxidative stress, carnosine

## Abstract

Age-related macular degeneration (AMD) has been described as a progressive eye disease characterized by irreversible impairment of central vision, and unfortunately, an effective treatment is still not available. It is well-known that amyloid-beta (Aβ) peptide is one of the major culprits in causing neurodegeneration in Alzheimer’s disease (AD). The extracellular accumulation of this peptide has also been found in drusen which lies under the retinal pigment epithelium (RPE) and represents one of the early signs of AMD pathology. Aβ aggregates, especially in the form of oligomers, are able to induce pro-oxidant (oxidative stress) and pro-inflammatory phenomena in RPE cells. ARPE-19 is a spontaneously arising human RPE cell line validated for drug discovery processes in AMD. In the present study, we employed ARPE-19 treated with Aβ oligomers, representing an in vitro model of AMD. We used a combination of methods, including ATPlite, quantitative real-time PCR, immunocytochemistry, as well as a fluorescent probe for reactive oxygen species to investigate the molecular alterations induced by Aβ oligomers. In particular, we found that Aβ exposure decreased the cell viability of ARPE-19 cells which was paralleled by increased inflammation (increased expression of pro-inflammatory mediators) and oxidative stress (increased expression of NADPH oxidase and ROS production) along with the destruction of ZO-1 tight junction protein. Once the damage was clarified, we investigated the therapeutic potential of carnosine, an endogenous dipeptide that is known to be reduced in AMD patients. Our findings demonstrate that carnosine was able to counteract most of the molecular alterations induced by the challenge of ARPE-19 with Aβ oligomers. These new findings obtained with ARPE-19 cells challenged with Aβ1-42 oligomers, along with the well-demonstrated multimodal mechanism of action of carnosine both in vitro and in vivo, able to prevent and/or counteract the dysfunctions elicited by Aβ oligomers, substantiate the neuroprotective potential of this dipeptide in the context of AMD pathology.

## 1. Introduction

Age-related macular degeneration (AMD) is a degenerative condition of the macula, the region of the central retina responsible for the greatest visual acuity, and represents the most common cause of irreversible blindness in elderly individuals due to the impairment of photoreceptor cells and retinal pigment epithelium (RPE) cells. The early stage of AMD is characterized by the accumulation of extracellular material, lipid, and protein aggregates between the RPE and Bruch’s membrane, lesions named drusen. Generally, AMD is classified into two forms: non-exudative form (dry) and exudative form (wet). The latter is characterized by choroidal neovascularization and blood-retinal barrier (BRB) breakdown induced by the overexpression of vascular endothelial growth factor (VEGF) [1]. Unfortunately, only palliative treatments are available for the wet form, including anti-VEGF antibodies, photodynamic therapy, and thermal laser therapy [2]. Currently, there are no pharmacological treatments for dry AMD; only oral supplementations with antioxidants are recommended.

Alzheimer’s disease (AD), the most common dementia in elderly patients [3], is often associated with AMD. In fact, vision-related alterations are common in AD, and visual defects are due to either degeneration of the visual cortex or to retinal degeneration associated with glaucoma and AMD. Several pieces of evidence indicate that the oligomeric form of amyloid-β (Aβ) peptide, one of the main actors in AD-related neurodegeneration, might be associated with AMD pathogenesis [4]. In particular, Aβ aggregates constitute the drusen deposits, resulting in chronic low-level inflammation and impairment of the retinal barrier [5]. Moreover, the accumulation of Aβ peptide in debris paralleled by the inflammatory processes could be considered a common pathogenetic mechanism linking these two neurodegenerative disorders [6]. Aβ deposits trigger a cascade of events activating microglia and retinal astrocytes with the secretion of pro-inflammatory cytokines, such as interleukin-1β (IL-1β), IL-6, and tumor necrosis factor-α (TNF-α), that, along with reactive oxygen species (ROS) formation, generate a harmful microenvironment, leading to retinal cells death and thinning of the retinal nerve fiber layer [7]. In addition, AD and AMD share similar pathophysiological features, including age, genetic factors, oxidative stress, and neuroinflammation [8,9].

Under physiological conditions, ROS are produced during oxidative metabolism, participating in basal cellular activity. However, when the amount of ROS exceeds the antioxidant system capability, ROS alter the balance of redox homeostasis, causing oxidative stress [10,11], which makes the retina susceptible to oxidative damage [12]. Several studies have shown that low levels of ROS can induce RPE cell apoptosis, while high levels of these species may trigger necrosis [13,14]. Several pieces of evidence report the link between oxidative stress and RPE dysfunction in AMD pathogenesis, so the identification of novel pharmacological targets and innovative neuroprotective strategies represents a crucial point [9].

In addition to neuroinflammation and oxidative stress phenomena, changes at the BRB level occur in an early phase of AMD pathogenesis. In fact, maintenance of physiological retinal cells structure, including RPE, requires tight junctions existence, such as zonula occludens (ZOs), responsible for molecular transport and essential for the BRB integrity, and its reduction or loss, under pathological conditions, increases barrier permeability [15].

Carnosine (β-alanyl-L-histidine) is an endogenous dipeptide distributed at high concentrations in the human central nervous system as well as in skeletal and cardiac muscles [16]. Numerous pre-clinical studies have shown the ability of carnosine to inhibit Aβ aggregation [17], to act as a scavenger of reactive species [18], and to exert anti-inflammatory activity by the modulation of immune cells [19,20,21]. With specific regard to AD, carnosine has shown neuroprotective activity in different in vitro models of Aβ-induced neuroinflammation and oxidative stress [22,23] as well as in animal models of AD [24], suggesting the important role of this natural dipeptide in preventing and/or counteracting degenerative disorders characterized by oxidative stress and neuroinflammation [25]. Moreover, in regard to AMD, it has been demonstrated that carnosine plasma levels are significantly reduced in AMD patients [26].

Based on the above, in the present study, we first investigated the toxic potential and molecular alterations induced by Aβ1-42 oligomers in ARPE-19, representing an in vitro model of AMD useful for drug-screening and/or biocompatibility testing of different molecules [1]. In particular, hereby we evaluated the modulation of inflammatory mediators, oxidative stress markers, and of ZO-1 protein expression after the Aβ1-42 oligomers challenge. Once the in vitro pathological model was characterized, we examined the therapeutic potential of carnosine in counteracting the enhancement of IL-1β, IL-6, TNF-α, and Nox-2 mRNA expression levels, the production of ROS, and the decrease of ZO-1 tight junction-associated protein levels.

## 2. Results

### 2.1. Aβ1-42 Oligomers Treatment Decreases Cell Viability and ATP Levels in ARPE-19 Cells

**Figure 1:**
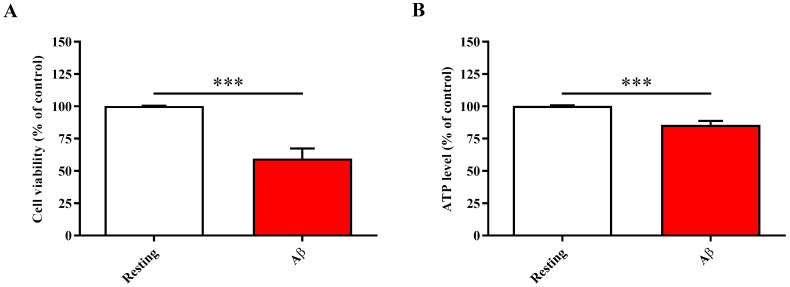
Change in (**A**) cell viability and (**B**) ATP intracellular levels caused by challenging ARPE-19 cells with Aβ1-42 oligomers. ARPE-19 cells were treated for 48 h with Aβ1-42 oligomers (2 µM). Data are the mean of four to seven values and are expressed as the percent variation with respect to the cell viability or ATP levels recorded in resting (control) cells. Standard deviations are represented by vertical bars. *** significantly different, *p* < 0.001.

Before examining the neuroprotective efficacy of carnosine, we first investigated the effects of Aβ1-42 oligomers on ARPE-19 cell viability and ATP intracellular content. As clearly shown in Figure 1A, the treatment of ARPE-19 cells with Aβ1-42 oligomers for 48 h significantly decreased cell viability compared to resting (control) cells (*p* < 0.001).

In line with the observed changes in cell viability, the treatment of ARPE-19 cells with Aβ1-42 oligomers significantly decreased ATP intracellular levels compared to resting (control) cells (*p* < 0.001) (Figure 1B), a molecular sign that cells are suffering and probably undergoing necrosis or apoptosis.

### 2.2. Aβ1-42 Oligomers Treatment Increases the Levels of Pro-Inflammatory and Pro-Oxidant Mediators

**Figure 2:**
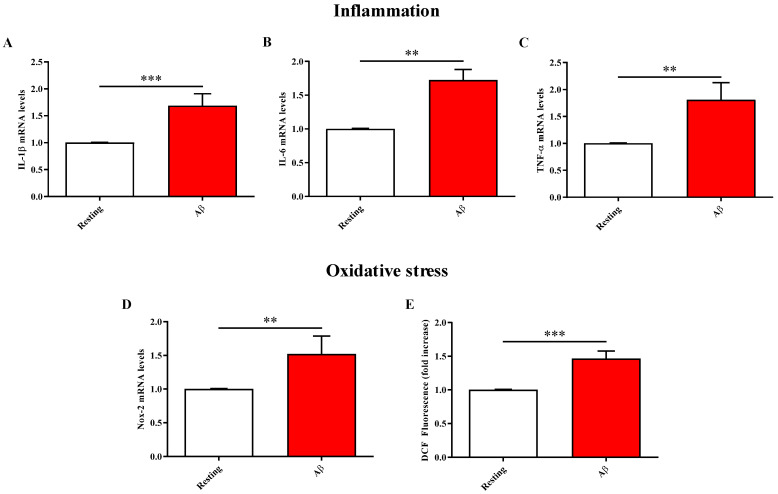
Effects of exposure of ARPE-19 cells to Aβ1-42 oligomers (2 µM) for 48 h on (**A**) IL-1β, (**B**) IL-6, (**C**) TNF α, (**D**) Nox-2 mRNA expression levels, and (**E**) intracellular ROS levels. The abundance of each mRNA of interest was expressed relative to the abundance of GAPDH, as an internal control. Production of ROS is expressed as fold increase with respect to the dichlorofluorescin (DCF) fluorescence measured in resting (control) cells. Values are reported as means of three to four values. Standard deviations are represented by vertical bars. ** significantly different, *p* < 0.01; *** significantly different, *p* < 0.001.

It is well-known the interplay between oxidative stress and inflammation in AMD pathogenesis, with the excess of ROS that can activate pro-inflammatory signaling pathways and the expression of multiple inflammatory mediators, such as cytokines, chemokines, and eicosanoids [27]. Based on this, we first investigated the effects of Aβ1-42 oligomers on mRNA expression levels of three well-known pro-inflammatory cytokines, namely IL-1β, IL-6, and TNF-α. As depicted in Figure 2A–C, the exposure of ARPE-19 cells to Aβ1-42 oligomers for 48 h led to a significant increase in mRNA expression levels of all the considered targets (*p* < 0.001 for IL-1β, *p* < 0.01 for IL-6 and TNF-α compared to resting cells).

Figure 2D,E also shows the ability of Aβ1-42 oligomers to induce oxidative stress, measured in terms of Nox-2 mRNAs expression levels and total ROS, in ARPE-19 cells. In fact, it was observed a significant upregulation of Nox-2 mRNA expression in ARPE-19 cells after 48 h exposure to Aβ1-42 oligomers (*p* < 0.01 compared to resting cells) (Figure 2D). As expected, this increase of Nox-2 mRNA expression Aβ-induced was paralleled by a significant enhancement in intracellular ROS levels (*p* < 0.001 compared to resting cells) (Figure 2E).

### 2.3. Aβ1-42 Oligomers Treatment Reduces the Expression Levels of ZO-1 Junction Protein

**Figure 3:**
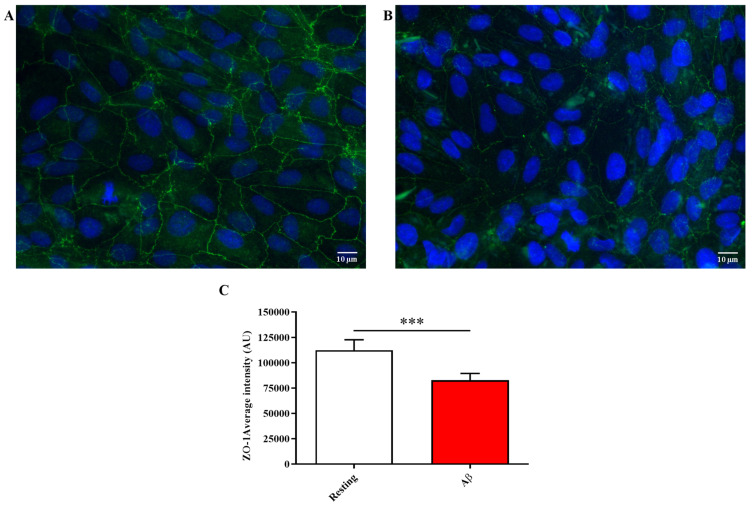
Immunocytochemistry evaluation of ZO-1 staining in (**A**) resting ARPE-19 cells and (**B**) ARPE-19 cells exposed to Aβ1-42 oligomers (2 µM) for 48 h. (**C**) The average intensity (AU) of the data from more than 10 values per coverslip for ZO-1 under our experimental conditions is shown. Standard deviations are represented by vertical bars. ZO-1 was labeled with FITC (green), while nuclei were labeled with 4′,6-diamidino-2-phenylindole (DAPI) (blue). *** significantly different, *p* < 0.001.

We then examined the impact of Aβ1-42 oligomers on ZO-1 tight junction protein expression, which plays a key role in maintaining BRB integrity. As shown in Figure 3, ZO-1 expression, measured as fluorescence arbitrary units (AUs), was significantly reduced after exposure for 48 h to Aβ1-42 oligomers compared to resting conditions (*p* < 0.001).

### 2.4. Carnosine Is Able to Counteract Most of the Molecular Alterations Induced by Aβ1-42 Oligomers in ARPE-19 Cells

Once the in vitro pathological model was characterized, the therapeutic potential of carnosine in counteracting the Aβ-associated molecular alterations was evaluated.

**Figure 4:**
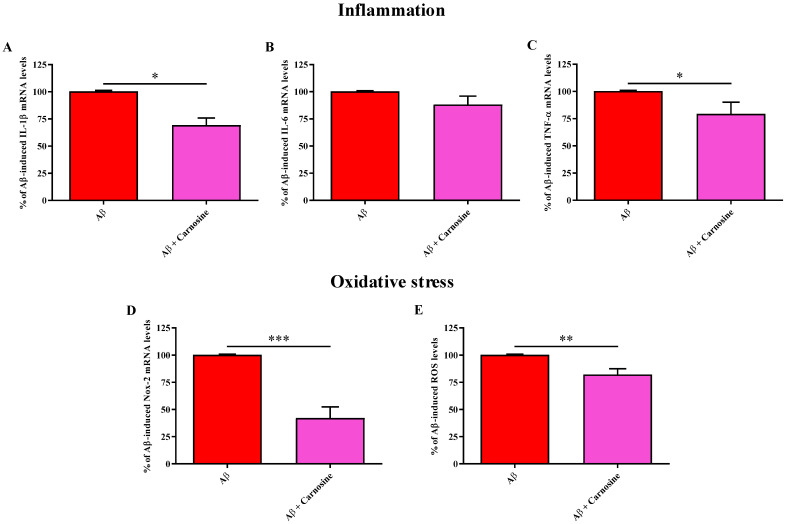
Effects of exposure of ARPE-19 cells to Aβ1-42 oligomers (2 µM) for 48 h, in the absence or presence of carnosine (20 mM; 1 h pre-treatment), on (**A**) IL-1β, (**B**) IL-6, (**C**) TNF-α (**D**) Nox-2 mRNA expression levels, and (**E**) intracellular ROS levels. The abundance of each mRNA of interest was expressed relative to the abundance of GAPDH, as an internal control. Production of ROS is expressed as fold increase with respect to the dichlorofluorescin (DCF) fluorescence measured in resting (control) cells. Values are reported as means of three values and are expressed as the percent variation with respect to IL-1β, IL-6, TNF-α, or Nox-2 mRNA expression levels or total ROS levels recorded in Aβ1-42 oligomers-treated cells. Standard deviations are represented by vertical bars. * significantly different, *p* < 0.05; ** significantly different, *p* < 0.01; *** significantly different, *p* < 0.001.

As reported in Figure 4, carnosine pre-treatment was able to significantly counteract the increase of mRNA expression levels of both IL-1β (Figure 4A) (*p* < 0.05) and TNF-α (Figure 4C) (*p* < 0.05) induced by Aβ1-42 oligomers, while no differences regarding IL-6 mRNA expression were observed in ARPE-19 cells exposed to Aβ1-42 oligomers for 48 h, in the absence or presence of carnosine (Figure 4B).

To further investigate the ability of carnosine to counteract the molecular alterations induced by Aβ1-42 oligomers, we then compared the mRNA expression of Nox-2 along with the intracellular ROS levels between ARPE-19 cells exposed to Aβ1-42 oligomers and ARPE-19 cells exposed to Aβ1-42 oligomers in the presence of carnosine.

Figure 4D shows the ability of carnosine to down-regulate the expression of Nox-2 ARPE-19 cells challenged with Aβ1-42 oligomers (*p* < 0.001). This effect was paralleled by carnosine’s ability to decrease the intracellular levels of ROS (Figure 4E) (*p* < 0.01).

**Figure 5:**
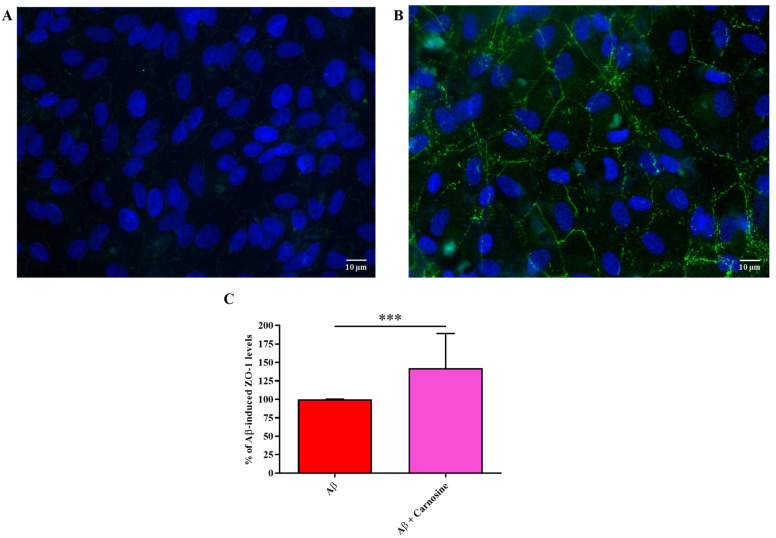
Immunocytochemistry evaluation of ZO-1 staining in ARPE-19 cells exposed to Aβ1-42 oligomers (2 µM) for 48 h in the (**A**) absence or (**B**) presence of carnosine (20 mM; 1 h pre-treatment). (**C**) The average intensity (AU) of the data from more than 10 values per coverslip for ZO-1 under our experimental conditions is shown and expressed as the percent variation with respect to ZO-1 expression levels in Aβ1-42 oligomers-treated cells. Standard deviations are represented by vertical bars. ZO-1 was labeled with FITC (green), while nuclei were labeled with DAPI (blue). *** significantly different, *p* < 0.001.

An additional protective activity of carnosine is shown in Figure 5. In fact, carnosine pre-treatment protected ARPE-19 cells against the Aβ-induced reduction of ZO-1 expression.

## 3. Discussion

AMD represents a multifactorial neurodegenerative and inflammatory disease primarily involving cellular and molecular components of the outer BRB; this barrier is damaged by complement fragments and RPE-derived factors, which stimulate immune cell activation, then promote an inflammatory response in the eye [28]. Of note, AD has been associated with AMD. In particular, it has been demonstrated that Aβ oligomers are involved in AMD pathogenesis [4,29], with the extracellular deposits of these species leading to the formation of drusen [5]. Additionally, AD and AMD share common pathophysiological features, including oxidative stress and neuroinflammation [8,9].

Carnosine is a naturally occurring endogenous dipeptide possessing a multimodal mechanism of action [30] that includes a well-recognized direct and indirect antioxidant activity [18], paralleled by anti-aggregation [31,32] and anti-inflammatory [33] effects. This suggests a potential therapeutic application of this dipeptide for the treatment of neurodegenerative disorders characterized by oxidative stress and inflammation, such as AMD [34,35,36]. Furthermore, carnosine plasma levels are significantly reduced in AMD patients [26], suggesting that a deficit of this peptide can contribute to AMD pathophysiology.

According to this scenario, in the present study, we first explored the toxic effects and molecular alterations induced by Aβ1-42 oligomers on ARPE-19 cells. It was observed that the treatment with Aβ1-42 oligomers significantly decreased the viability of ARPE-19 (Figure 1A), also decreasing the ATP levels (Figure 1B). These Aβ oligomers’ toxic effects were paralleled by a significant enhancement of the oxidative stress, measured as the induction of Nox-2 pro-oxidant enzyme mRNA expression levels and ROS production (Figure 2D,E). Moreover, the inflammatory process was exacerbated, as underlined by the up-regulation of the expression of IL-1β, IL-6, and TNF-α cytokines (Figure 2A–C), with the direct consequence of a significant decrease of ZO-1 tight junction-associated protein levels (Figure 3). The above-described results are in line with the deleterious effects of the oligomeric forms of Aβ1-42 peptide, representing the most toxic species of Aβ [37,38]. In fact, numerous studies have shown that these oligomers are able to lead to synaptic loss and neuronal death [39]. Aβ toxic effects can be mediated by the induction of both neuroinflammation, through the production of pro-inflammatory mediators [40], and oxidative stress; in fact, Aβ oligomers have been shown to promote neurodegeneration and neuroinflammation via oxidative stress [41,42]. It has also been demonstrated that oxidative stress promotes the oligomerization of Aβ peptide [43], making the peptide highly neurotoxic. With specific regard to ARPE-19, oligomeric Aβ1-42 can trigger AMD-like injury by activating poly(ADP-ribose) polymerase (PARP1) and repressing Sirtuin (SIRT1) [44], while a different study carried out by Varinthra et al. showed elevated expression of TNF-α, cyclooxygenase-2, and inducible nitric oxide synthase via nuclear factor kappa-light-chain-enhancer of activated B cells signaling [45]. Our work contributes to identifying, in an experimental model of AMD, the key role of IL-1β and TNF-α combined with oxidative stress in ARPE-19 cell degeneration.

Once the in vitro pathological model was characterized, we then examined the therapeutic potential of carnosine in counteracting the deleterious effects induced by Aβ1-42 oligomers in ARPE-19 cells.

Carnosine was able to counteract almost all the molecular alterations induced by Aβ1-42 oligomers in ARPE-19 cells (Figure 4 and Figure 5). In particular, carnosine pre-treatment was able to significantly decrease the mRNA expression levels of both IL-1β and TNF-α (Figure 4A,C), which exert a central role in initiating the inflammatory process. Several studies have linked the deleterious effects of IL-1β with different pathological conditions, such as diabetes [46], AD [47], and AMD [48]. It has also been shown that high extracellular levels of TNF-α are linked to the worsening of pro-inflammatory and neurodegenerative phenomena [49,50]. Our data demonstrate for the first time a relevant neuroprotective role of carnosine in counteracting inflammatory phenomena in the context of AMD and are in accordance with the anti-inflammatory activity showed by this dipeptide in different models of neurodegenerative disorders [51].

In our experimental AMD model, carnosine was also able to decrease oxidative stress as assessed by Nox-2 pro-oxidant enzyme mRNA expression levels and total ROS production. Both Nox-2 expression and ROS production increased as a consequence of the Aβ1-42 oligomers challenge and were significantly diminished in the presence of carnosine (Figure 4D,E). These findings are in line with the well-recognized antioxidant activity of carnosine linked with its ability to interact directly with these species [52] and the presence of the imidazole ring part of histidine amino acid [53]. Our results, showing the ability of this dipeptide to reduce species related to oxidative stress phenomena, are also in accordance with other studies in which carnosine protected neuronal cells against oxidative stress via the modulation of mitogen-activated protein kinase pathway [54] or exerted neuroprotection in primary cells exposed to treatments able to induce oxidative stress by generating free radicals [55]. The observed decrease in intracellular ROS levels could also depend on the increased loading of carnosine under stress conditions [21], the ability of carnosine to increase the rate of conversion of reactive mediators into their non-toxic end-products [53], and/or of the ability of carnosine to preserve the monomeric form of Aβ peptide or to disassemble the Aβ oligomers already formed [56,57]. We cannot exclude that carnosine can exert its protective effects on ARPE-19 cells also through other mechanisms, e.g., promoting the release of neurotrophic factors such as transforming growth factor-β1 known to be reduced both in AMD patients and experimental models of AMD [58,59,60]. Further studies are therefore needed to explore the neuroprotective efficacy of carnosine in experimental models of AMD.

Lastly, we were able to demonstrate as carnosine pre-treatment protected ARPE-19 cells against the reduction of ZO-1 expression Aβ-induced, preserving and/or counteracting the deleterious effects exerted by Aβ1-42 oligomers (Figure 5). This result is particularly important considering that RPE-barrier dysfunction has also been associated with attenuation/disruption of ZO-1 [61].

All the above-described results, describing the ability of carnosine to counteract the molecular alterations observed in ARPE-19, are relevant for drug discovery processes in AMD since it is known that RPE, a single-cell layer at the posterior part of the eye, plays a significant role in the pathogenesis of AMD. In healthy conditions, RPE cells are responsible for maintaining the functionality of the overlying photoreceptor cells, protection of the retina from excessive light, formation of the BRB in conjunction with the vascular endothelium, and immune defense of the macula [62]. A functional degeneration of the RPE leads to impaired maintenance of the sensory retina, which contributes to vision loss in advanced AMD. Despite these promising results obtained by using carnosine, further preclinical studies are needed in order to translate these findings into in vivo and clinical studies.

The therapeutic relevance of carnosine in the context of AMD pathology recently emerged in a clinical study conducted by Chao de la Barca and collaborators [26]. In this study, in which the plasma metabolomic profile of exudative was determined in 40 AMD patients and 40 age- and sex-matched subjects, carnosine was the only metabolite showing a significantly reduced concentration in the AMD group with an almost half the mean concentration compared to controls, demonstrating for the first time a carnosine deficiency in AMD. Since increased oxidative stress, as well as the formation of advanced glycation end products, have been observed in AMD retina [6], this study suggests that the relative deficiency in carnosine could contribute to AMD pathogenesis and thus open a novel path for drug development and possible therapeutic perspectives.

## 4. Materials and Methods

### 4.1. Materials and Reagents

All materials and reagents were of analytical grade and supplied by Sigma-Aldrich (St. Louis, MO, USA) or Thermo Fisher Scientific (Waltham, MA, USA) unless differently specified. ARPE-19 (human retinal pigment epithelial) cells (ATCC^®^ CRL-2302™), DMEM:F12 medium, fetal bovine serum (FBS), trypsin-EDTA solution, and penicillin/streptomycin solution were purchased from American Type Culture Collection (ATCC, Manassas, VA, USA). HFIP-treated amyloid β-peptide (1-42) and Amyloid β-Protein (42-1) were obtained from Bachem Distribution Services GmbH (Weil am Rhein, Germany). C-Chip disposable hemocytometers, used for ARPE-19 cell counting, were obtained from Li StarFish S.r.l. (Naviglio, Italy). ATPlite 1 step kit was supplied by Perkin Elmer (Monza, Italy). QuantiTect SYBR Green PCR Kits and QuantiTect Primer Assays were purchased from Qiagen (Hilden, Germany). The 384-well plates were obtained by Roche Molecular Systems Inc. (Pleasanton, CA, USA). Eppendorf LoBind 1.5 mL Microcentrifuge Tubes PCR Clean as well as PCR tubes were obtained from Eppendorf (Hamburg, Germany).

### 4.2. Preparation of Aβ1-42 Oligomers and Selection of Carnosine Concentration

The preparation of Aβ1-42 oligomers was achieved by employing a well-validated protocol previously described in detail [63]. Briefly, the HFIP-treated Aβ1-42, lyophilized and under the monomeric form, was suspended in dimethyl sulfoxide at the final concentration of 5 mM. Ice-cold DMEM/F12 (1:1) medium was instead used to further dilute (100 μM) all the samples. Aβ1-42 samples were then incubated for 48 h at 4 °C, at the end of which the formed oligomers were immediately used to treat ARPE-19 cells or aliquoted and stored at −20 °C until their use. Atomic force microscopy (AFM) was previously used to assess the suitability of this method used to obtain the formation of Aβ1-42 oligomers [64]. Preliminary experiments by employing Aβ42-1 (reverse sequence of Aβ1-42, inactive control for the Aβ) demonstrated no effects on the modulation of cell viability, the expression of IL-6, TNF-α, and IL-1β mRNAs, as well as on ROS production compared to untreated ARPE-19 cells.

### 4.3. Propagation and Maintenance of cells

The authentication of the cell line used in this study (ARPE-19) was performed by Eurofins Genomics Europe Applied Genomics GmbH (Ebersberg, Germany) [65] (Supplementary material: Supplementary Files S1–S5). ARPE-19 cells were cultured in DMEM:F12 medium enriched with FBS (10%), streptomycin (100 μg/mL), and penicillin s1 (100 U/mL) by using 25 or 75 cm^2^ polystyrene culture flasks. Cells were maintained in a humidified environment (37 °C, 5% CO_2_). In order to avoid cell overgrowth, ARPE-19 cells were passaged every 2–3 days.

### 4.4. Analysis of Cell Viability

The appropriate concentration of Aβ1-42 oligomers able to exert toxic effects in ARPE-19 cells was selected by preliminary testing three different Aβ concentrations (0.5, 1, 2 μM), while carnosine was used at the concentration of 20 mM, representing the gold standard in in vitro studies [16,21,66,67,68,69], a selection also sustained by preliminary experiments. In particular, we first tested the effects of increasing concentrations of carnosine on ARPE-19 cell viability (Figure S1). By doing so, we were able to select the highest concentration (20 mM) that could be used in this specific cell line without significant changes in cell viability, which was also more effective in preventing the toxic effects induced by Aβ oligomers (Figure S2). 

ARPE-19 cells were harvested (trypsin-EDTA solution), counted (C-Chip disposable hemocytometer), and plated in 96-well plates (1.5 × 10^4^ cells/well). The day after, cells were treated with Aβ1-42 oligomers (2 μM) and incubated for 48 h in a humidified environment (37 °C, 5% CO_2_). At the end of the stimulation process, cell viability was measured by employing the MTT (3-[4,5-dimethylthiazol-2-yl]-2,5 diphenyl tetrazolium bromide) assay as previously described [70].

The effects of Aβ1-42 oligomers on ARPE-19 cell status were also evaluated by measuring the ATP production with the ATPlite 1 step kit according to the manufacturer’s instructions [71]. The concentration of ATP will be proportional to the luminescence intensity coming from its reaction with luciferase and D-luciferin. At the end of the treatment, the plate was equilibrated at room temperature and added to the reaction solution. The luminescence was then measured with a Varioskan^®^Flash spectrophotometer (Thermo Fisher Scientific, Waltham, MA, USA).

### 4.5. Gene Expression Analysis by Quantitative Real-Time PCR (qRT-PCR)

Extraction of total RNA from ARPE-19 cells was performed with a TRIzol Reagent. The concentration of total RNA recovered from untreated ARPE-19 cells or cells treated with Aβ1-42 oligomers (2 μM), in the absence or presence of carnosine (20 mM), for 48 h was determined through NanoDrop^®^ ND-1000 (Thermo Fisher Scientific, Waltham, MA, USA), by measuring the absorbance at 260 nm; Qubit^®^ 3.0 Fluorometer (Thermo Fisher Scientific) was instead used to test RNA quality [72]. cDNA was synthesized from 2 μg of RNA with a reverse transcription kit (SuperScript™ II Reverse transcriptase) according to manufacturer instructions. The quantification of cDNA samples loaded in a 384-well plate was obtained by employing a LightCycler^®^ 480 System (Roche Molecular Systems, Inc., Pleasanton, CA, USA). Table 1 reports the information related to the genomewide, bioinformatically validated primer sets (QuantiTect Primer Assays) employed for the gene expression analysis. 

The protocol used to perform sample amplification, fluorescence data collection, as well as sample quantification is the same as previously described [73]. The selected housekeeping reference gene was GAPDH.

### 4.6. Immunohistochemistry

Immunocytochemistry analysis of ZO-1 was carried out as previously described [15]. Briefly, after a washing step with phosphate-buffered saline (PBS), ARPE-19 were fixed with ice-cold acetone and incubated with ice-cold methanol. Cells permealization was obtained by using a solution consisting of PBS, normal goat serum, and Triton-X 100, followed by the incubation with ZO-1 antibody (1:100). After PBS washings, ARPE-19 cells were incubated with FITC-conjugated goat anti-rabbit antibody (1:300), while nuclei were marked with DAPI (1:10,000). The semi-quantitative evaluation of ZO-1 expression levels was carried out as previously described [15,74]. Briefly, coverslips were mounted on glass slides through the use of a mounting medium and analyzed by using an epifluorescent Zeiss Observer Z1 microscope equipped with the Apotome.2 acquisition system connected to a digital camera (Carl Zeiss Microscopy GmbH, Oberkochen, Germany). ZO-1 immunostaining images were analyzed with ImageJ software [75].

### 4.7. Measurement of ROS Production

The ability of carnosine to counteract the changes in intracellular ROS levels due to Aβ oligomers treatment for 48 h was carried out in ARPE-19 cells by using a 2′,7′-dichlorofluorescin diacetate (DCFDA) cellular ROS assay kit, according to the manufacturer’s recommendations. ROS quantification was achieved by measuring the fluorescence (excitation = 485 nm; emission = 535 nm) with a Varioskan Flash microplate reader (Thermo Fisher Scientific) and normalized to the fluorescent intensity of untreated ARPE-19 cells (control).

### 4.8. Statistical Analysis

Statistical data analysis was carried out by using version 8.0 of the software Graphpad Prism (GraphPad software, San Diego, CA, USA). Student’s *t*-test was used to assess the statistical differences between the two experimental groups. Only *p*-values of less than 0.05 were considered statistically significant. Data are always reported as the mean ± SD of at least three values.

## 5. Conclusions

In the present study, we were able to show that carnosine suppresses oxidative stress and inflammation induced by Aβ1-42 oligomers in ARPE-19 cells. In particular, this dipeptide decreased ROS levels and the mRNA expression of pro-oxidant and pro-inflammatory mediators, i.e., Nox-2, IL-1β, and TNF-α. Moreover, carnosine protected ARPE-19 cells against Aβ1-42 oligomers-induced BRB impairment, as evidenced by ZO-1 protein immunostaining. Our results suggest a neuroprotective potential of carnosine in this in vitro model, a translational and validated paradigm of AMD disease.

## Figures and Tables

**Table 1:** The list of primers used for qRT-PCR.

Official Name ^#^	Official Symbol	Alternative Titles/Symbols	Detected Transcript	Amplicon Length	Cat. No. ^§^
interleukin 1, beta	IL1B	IL-1; IL1F2; IL1beta; IL1-BETA	NM_000576; XM_006712496	117 base pair (bp)	QT00021385
interleukin 6	IL6	CDF; HGF; HSF; BSF2; IL-6; BSF-2; IFNB2; IFN-beta-2	NM_000600; XM_005249745	107 bp	QT00083720
tumor necrosis factor	TNF	DIF; TNFA; TNFSF2; TNLG1F; TNF-alpha	NM_000594	98 bp	QT00029162
cytochrome b-245 beta chain	CYBB	CGD; CGDX; NOX2; IMD34; AMCBX2; GP91-1; GP91PHOX; p91-PHOX; GP91-PHOX	NM_000397	124 bp	QT00029533
glyceraldehyde-3-phosphate dehydrogenase	GAPDH	G3PD; GAPD; HEL-S-162eP	NM_001256799; NM_002046; NM_001289745; NM_001289746	95 bp	QT00079247

^#^ https://www.ncbi.nlm.nih.gov/gene/ (accessed on 3 January 2023); ^§^ https://www.qiagen.com/it/shop/pcr/real-time-pcr-enzymes-and-kits/two-step-qrt-pcr/quantitect-primer-assays/ (accessed on 3 January 2023).

## Data Availability

The data presented in this study are available on request from the corresponding author.

## Supplementary Materials

The following supporting information can be downloaded at: https://www.mdpi.com/article/10.3390/molecules28083324/s1. Figure S1: Cell viability in resting ARPE-19 cells and in ARPE-19 cells treated with increasing concentrations of carnosine (1, 10, 20, 50, 100, and 200 mM) for 48 h assessed by MTT assay. Figure S2: Cell viability in resting ARPE-19 cells and in ARPE-19 cells treated with Aβ1-42 oligomers (2 µM) in the absence or presence of increasing concentrations of carnosine (1 and 20) for 48 h assessed by MTT assay. Supplementary Files S1–S5.
