# Supplementary material for: Carnosine Counteracts the Molecular Alterations Aβ Oligomers-Induced in Human Retinal Pigment Epithelial Cells
Source: Molecules. 2023 Apr 9;28(8):3324. doi: 10.3390/molecules28083324 (PMC10146178; doi:10.3390/molecules28083324)
Supplement: Supplementary file 1 [file molecules-28-03324-s001.zip › Supp file 1.pdf]

Eurofins Genomics Europe Applied Genomics GmbH, Anzinger Str. 7 a, D-85560 Ebersberg

Dr. Nicolò Musso
University of Catania, BIOMETEC
Via Santa Sofia 97
95124 Catania
Italy

eurofins | Genomics

SOP_APG_Zelllinienauthentizität_A04_2.0

Person in charge: Dr. Torsten Brendel
Report date: 29.03.2023
Sample received on: 22.03.2023
Start / End of Analysis: 22.03.2023/29.03.2023

Analytical Report:
Cell Line Authentication Test
Order ID: 11108356895

Method:

Genetic characteristics were determined by PCR-single-locus-technology.
16 independent PCR-systems D8S1179, D21S11, D7S820, CSF1PO, D3S1358, TH01, D13S317, D16S539, D2S1338, AMEL, D5S818, FGA, D19S433, vWA, TPOX and D18S51 were investigated.
(ASN-0002 core markers are colored grey, Thermo Fisher, AmpFlSTR® Identifiler® Plus PCR Amplification Kit)
In parallel, positive and negative controls were carried out yielding correct results.
Method details are given in **SOP_APG_Zelllinienauthentizität_2.0**

Result:

**Client Sample Name**	ARPE-19
**Sample Code**	CL00012416
D8S1179	13,13
D21S11	28,29
D7S820	9,11
CSF1PO	11,11
D3S1358	14,15
TH01	6,9.3
D13S317	11,12
D16S539	9,11
D2S1338	19,19
D19S433	12,13
vWA	16,19
TPOX	9,11
D18S51	12,16
AMEL	X,Y
D5S818	13,13
FGA	23,23
**Database Name**	ARPE-19

The table shows the result of the cell line analysis and the comparison with the online database of the DSMZ (http://www.dsmz.de/de/service/services-human-and-animal-cell) and the Cellosaurus database (https://web.expasy.org/cellosaurus). Please note that only the PCR-systems according to ANSI/ATCC standard ASN-0002 were aligned (D5S818, D13S317, D7S820, D16S539, VWA, TH01, TPOX, CSF1PO, AMEL - colored grey).

The laboratory is accredited acc. to **DIN EN ISO/IEC 17025:2018**. All analyses have been carried out with greatest care and on the basis of state of the art scientific knowledge. The results refer solely to the analysed samples, as received. The duplication and publication also in parts requires a written authorization by this laboratory. Our General Terms and Conditions apply exclusively and are available under eurofinsgenomics.com

ilac-MRA DAkkS Deutsche Akkreditierungsstelle D-PL-13372-01-00

This report was created automatically and is therefore valid without a signature.

Eurofins Genomics Europe Applied Genomics GmbH
Anzinger Straße 7 a
85560 Ebersberg
Germany

Tel.: +49 7531 816068
Email: support-eu@genomics.eurofinseu.com
Web: www.eurofinsgenomics.com

Managing Director: Dr. Michael Hadem
Authorized Officer: Matthias Dittmer

Register Court Munich HRB 207710
VAT ID: DE815473648

HypoVereinsbank
IBAN: DE23 2073 0017 7000 0006 50
SWIFT: HYVEDEMME17
