# Supplementary figures and images for: Carnosine Counteracts the Molecular Alterations Aβ Oligomers-Induced in Human Retinal Pigment Epithelial Cells
Source: Molecules. 2023 Apr 9;28(8):3324. doi: 10.3390/molecules28083324 (PMC10146178; doi:10.3390/molecules28083324)

## Slide 1
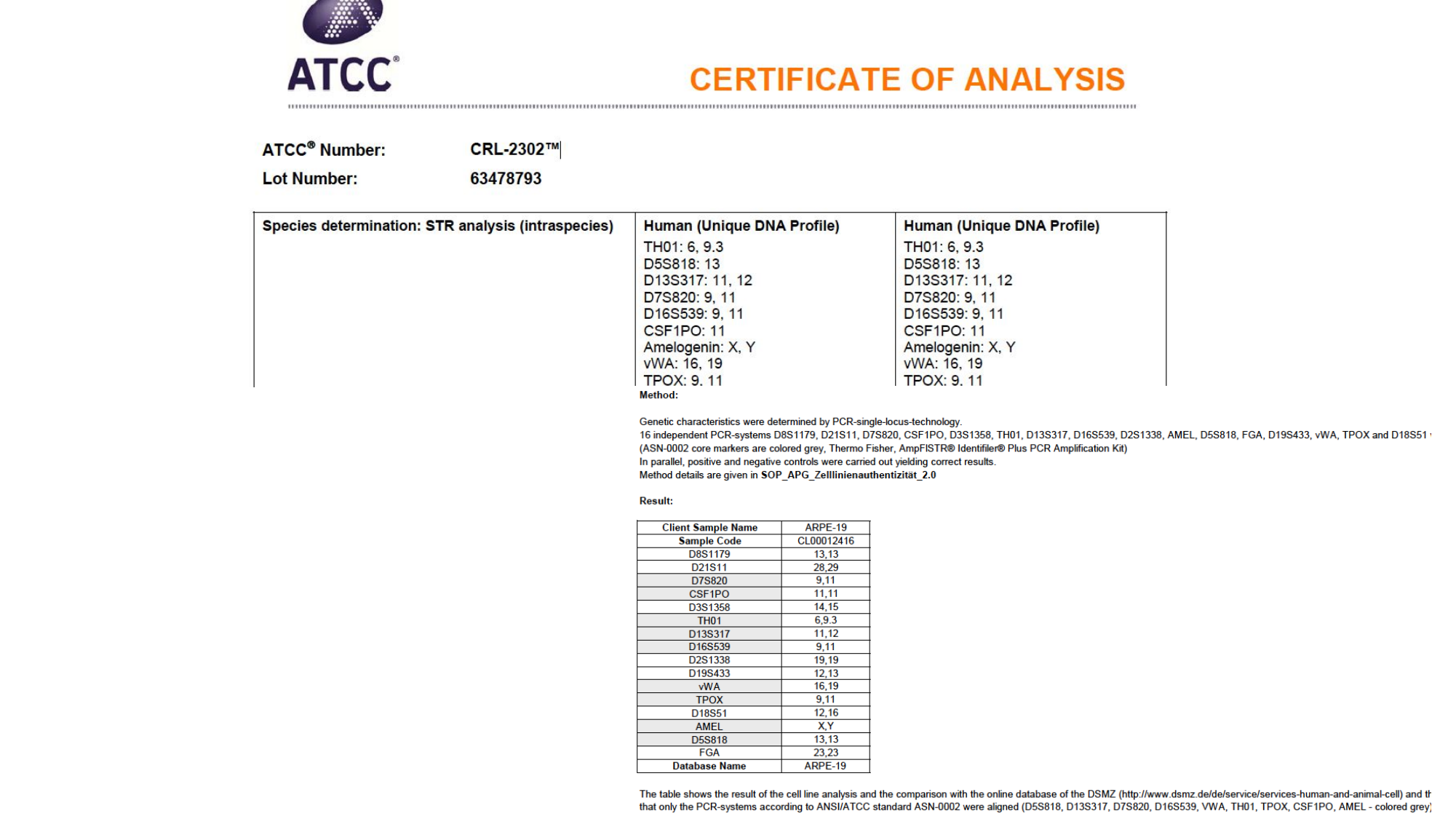

Supplement: Supplementary file 1 [file molecules-28-03324-s001.zip › Data.pptx]

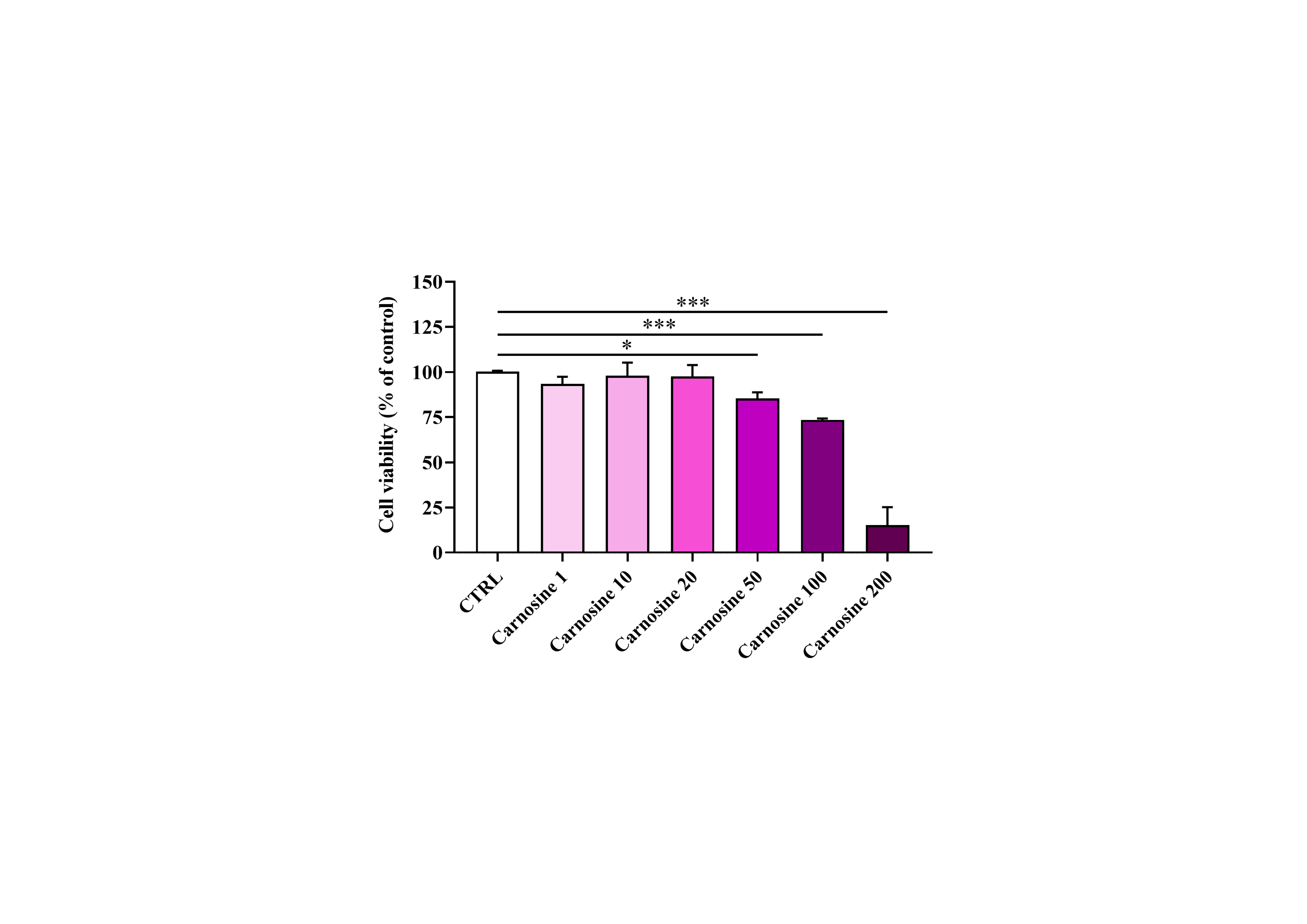

Supplement: Supplementary file 1 [file molecules-28-03324-s001.zip › Figure S1.tif]

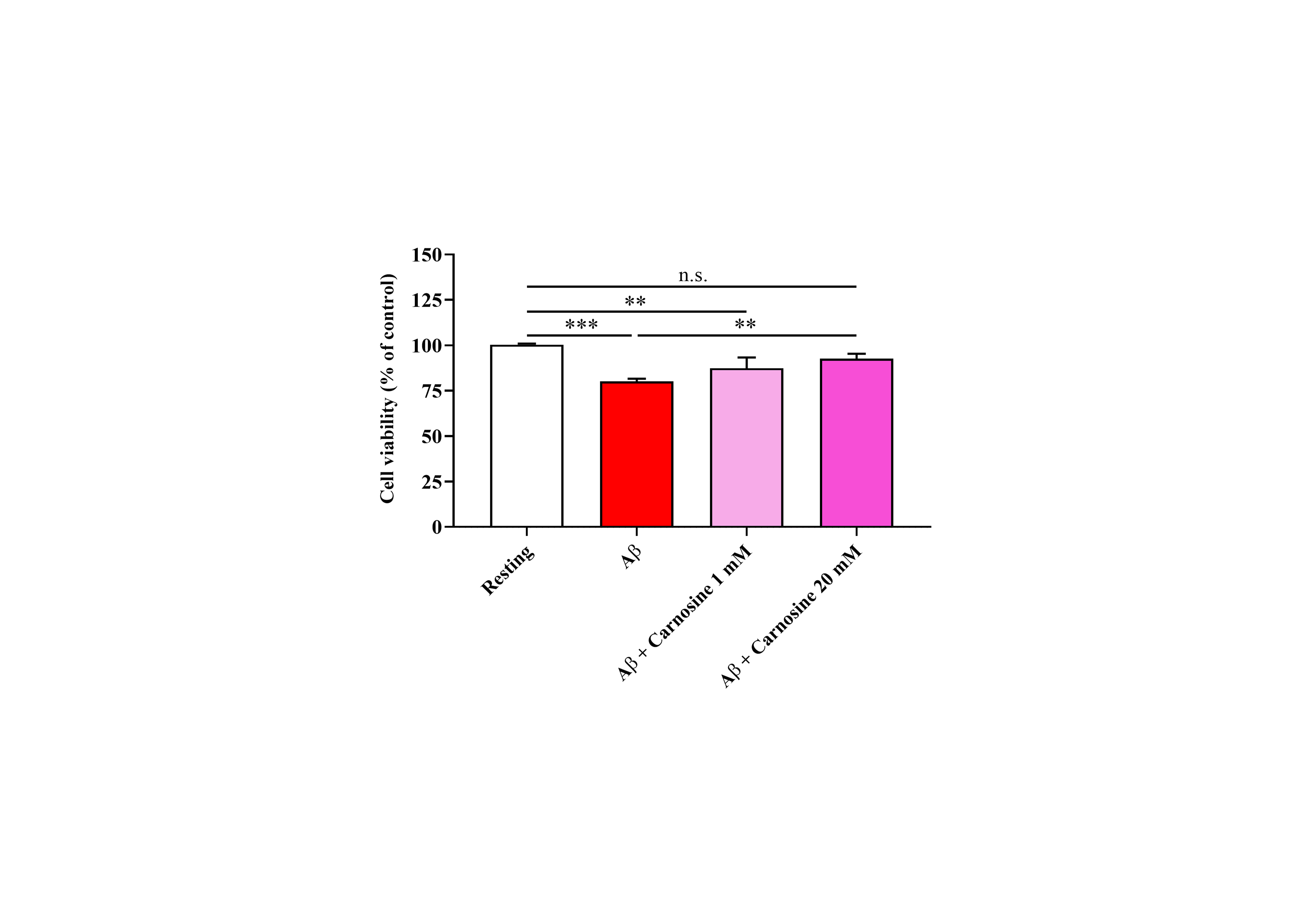

Supplement: Supplementary file 1 [file molecules-28-03324-s001.zip › Figure S2.tif]

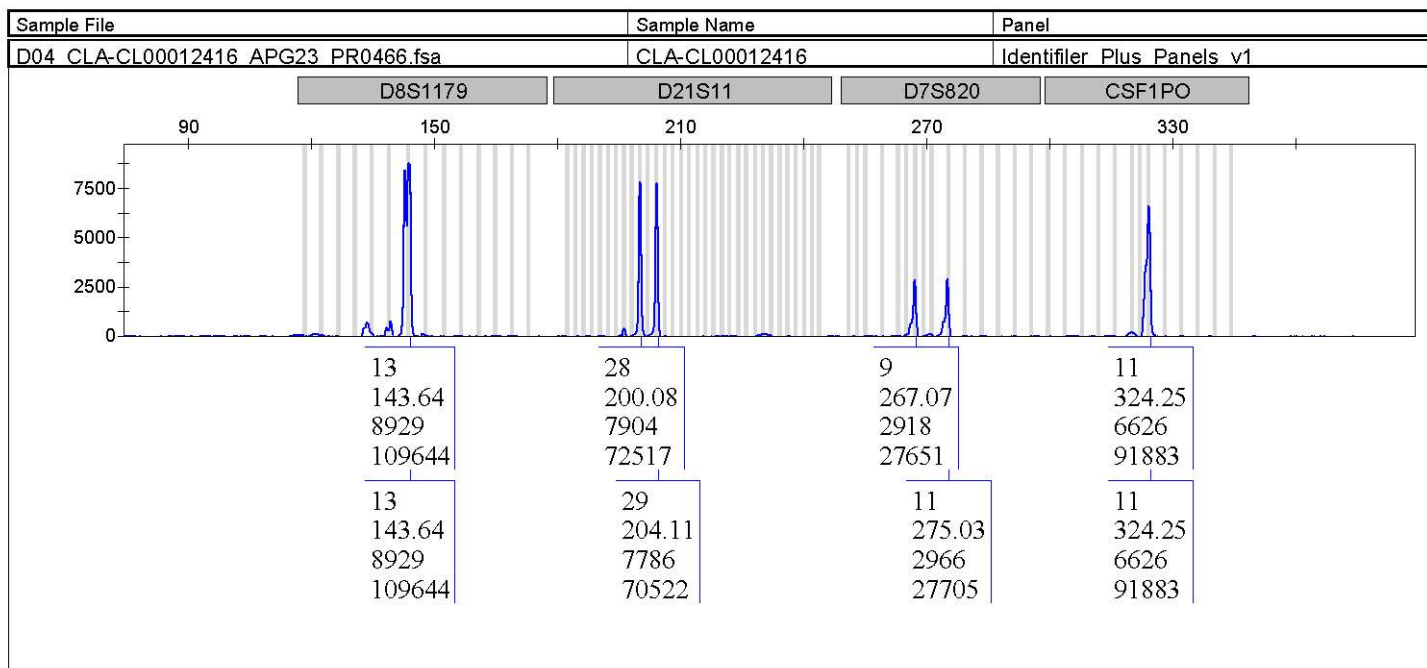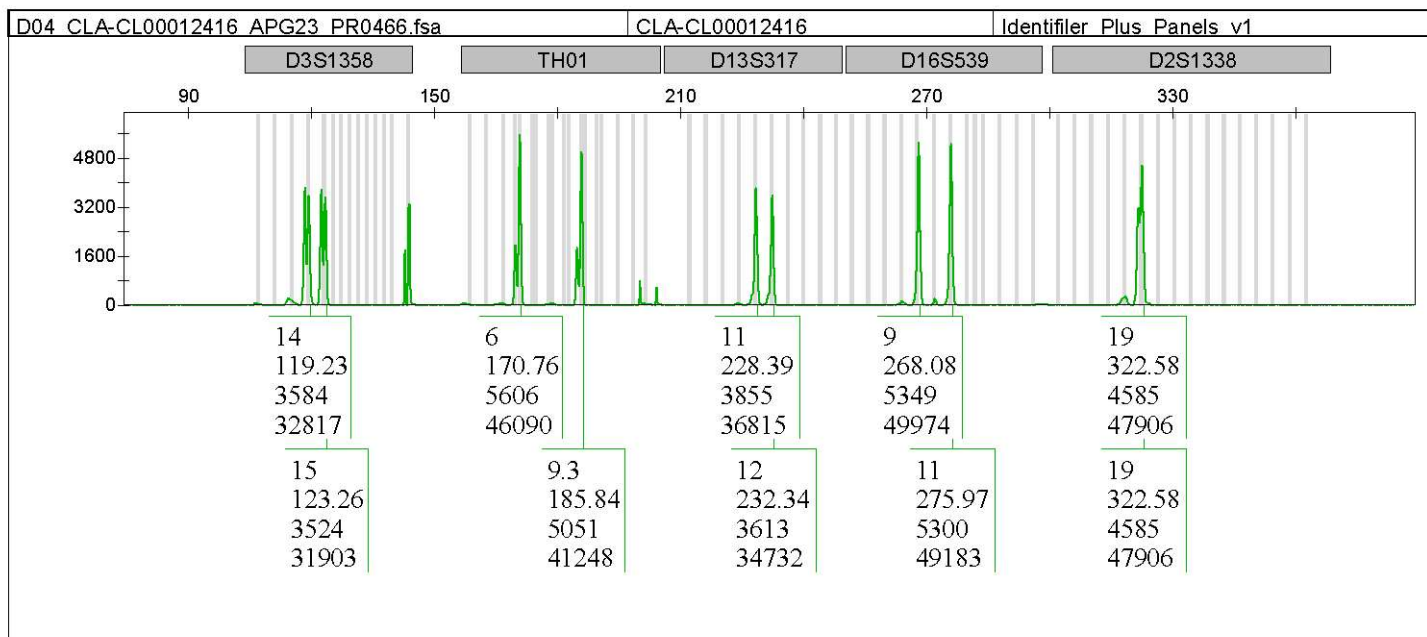

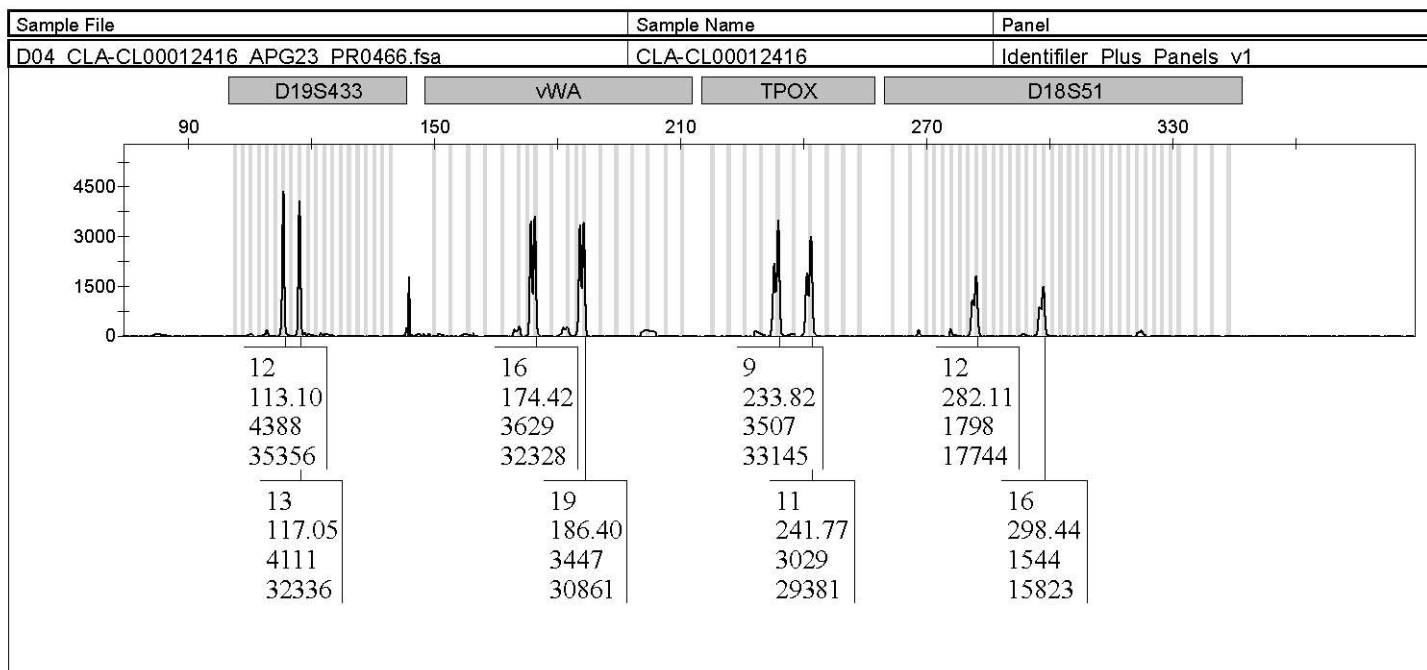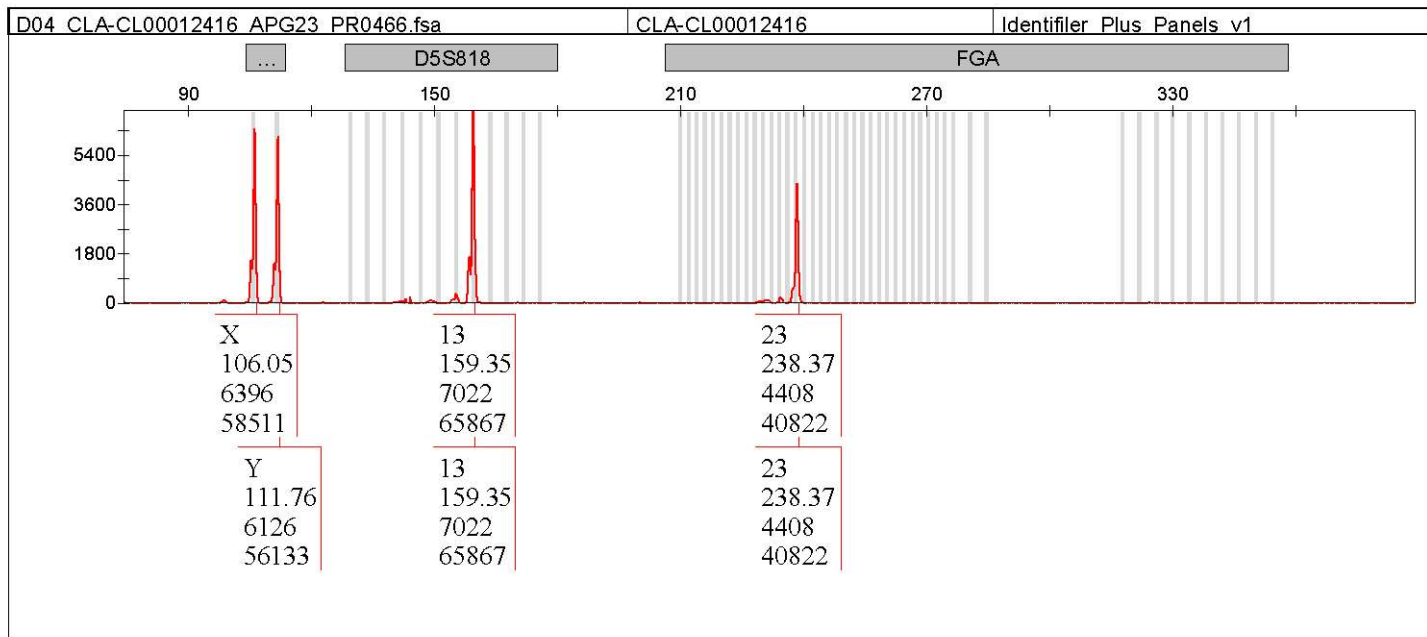

Supplement: Supplementary file 1 [file molecules-28-03324-s001.zip › Supp file 2.pdf]
